# The role of menstrual cycle phase and AMH levels in breast cancer patients whose ovarian tissue was cryopreserved for oncofertility treatment

**DOI:** 10.1007/s10815-014-0392-z

**Published:** 2014-12-03

**Authors:** Seido Takae, Yodo Sugishita, Nobuhito Yoshioka, Mariko Hoshina, Yuki Horage, Yorino Sato, Chie Nishijima, Kazuhiro Kawamura, Nao Suzuki

**Affiliations:** Department of Obstetrics and Gynecology, St. Marianna University School of Medicine, Kawasaki City, Kanagawa prefecture Japan 216-8511

**Keywords:** Combined procedure, Ovarian tissue cryopreservation, Oncofertility, Fertility preservation, In virto maturation, Breast cancer

## Abstract

**Purpose:**

To determine the factors that affect oocyte extraction efficiency when using the “combined procedure”. In the present “combined procedure” ovarian tissue cryopreservation and oocyte extraction from an isolated ovary, later used in In Vitro Maturation (IVM), are performed concurrently.

**Methods:**

Data were analyzed retrospectively and obtained from the clinical records of 27 young breast cancer patients referred for fertility preservation.

**Results:**

The patients’ mean age was 33.7 (±3.8) years, mean serum anti-Müllerian hormone (AMH) concentration was 3.5 (±2.1) ng/ml, and mean number of extracted oocytes was 8.3 (±6.1). The phase of menstruation (follicular or luteal) did not affect either the number of oocytes extracted (*P* = 0.99) nor oocyte survival or maturation rates. Likewise, the number of oocytes that could be extracted was not affected by the type of laparoscopic procedure (multiple-port or single-incision laparoscopy; *P* = 0.94) or the molecular subtype of breast cancer (either Luminal A or B; *P* = 0.52). Analysis revealed that the number of extracted oocytes was well-correlated with the patient’s AMH serum level and age (coefficient of correlation: 0.60 and −0.48, respectively).

**Conclusion:**

We conclude that the outcome of the “combined procedure” primarily depends upon the patient’s serum AMH level and age. Importantly, the “combined procedure” may be used during any phase of the menstrual cycle to preserve the fertility of breast cancer patients.

## Introduction

The number of young breast cancer patients who desire to bear children has increased in Japan [1]. This trend is likely due to the increasing incidence of breast cancer and the tendency of young women to marry later. Therefore, fertility preservation for young breast cancer patients has become a significant medical concern. Although it is not easy to assess the degree of gonadal damage, the level of ovarian dysfunction depends upon the patient’s age, the type and regimen of chemotherapy, and drug dosage. Chemotherapy for the treatment of breast cancer may induce amenorrhea or premature ovarian failure (POF) [2]. In breast cancer patients aged >40 years, the front line chemotherapy regimen AC or AC-T (anthracycline and cyclophosphamide, and taxane) could decrease ovarian reserve. Although CMF (cyclophosphamide and methotrexate and 5-FU) is not first line treatment, it could induce POF for more than half of breast cancer patients aged 30–39 years. In addition, the effect of taxane on ovarian reserve is still controversial. Of the 242 breast cancer patients who were administered AC or trastuzumab and paclitaxel, paclitaxel did not increase the risk of amenorrhea [3]. However, the increased risk of POF caused by taxane has been considered [4].

For breast cancer patients who are at risk for POF, due to chemotherapy, there are three options for fertility preservation. According to the 2nd guideline of the American Society of Clinical Oncology (ASCO) for fertility preservation, embryo and oocyte cryopreservation are established methods for fertility preservation; however, the third option of ovarian tissue cryopreservation is experimental and not yet an established method [5]. Although embryo and oocyte cryopreservation are established methods, many cancer patients cannot utilize these options because either oocyte extraction would require delaying chemotherapy or because their cancer is an estrogen-dependent type. FertiPROTEKT suggests ovarian tissue cryopreservation as a fertility preservation option for cancer patients who have too short a time (less than two weeks) before chemotherapy for embryo and/or oocyte cryopreservation [6]. At present, there have been around 30 live births from transplanted ovarian tissue. In addition, Kawamura et al. recently investigated the vitrification methods of ovary tissue cryopreservation and reported two cases of pregnancy using oocytes from vitreous-warmed ovarian tissue [7, 8]. Ovarian tissue cryopreservation is advantageous for breast cancer patients because they may be able to reserve many follicles within a few days. Furthermore, some researchers have reported the efficacy of a combined procedure in which various fertility preservation methods are combined [9–11]. In the present “combined procedure” ovarian tissue cryopreservation and oocyte extraction from an isolated ovary, later used IVM, are performed concurrently.

In present study, we seek to elucidate the factors that correlated to the efficacy of “combined procedure” on young breast cancer patients who received ovarian tissue cryopreservation for fertility preservation.

## Materials and methods

### Patients

Data were retrospectively obtained from the clinical records of breast cancer patients who were referred to the Oncofertility Unit at the Center for Reproductive Medicine, Department of Obstetrics and Gynecology of our university hospital from February 2010 to March 2014. In the current study, all patients gave written, informed consent in keeping with the Declaration of Helsinki, and the study was approved by the institutional review board of university. We decided the procedure of fertility preservation (oocyte and embryo cryopreservation, or ovary tissue cryopreservation) according to period until chemotherapy and age of patient. Mostly patients who received ovary tissue cryopreservation had not enough time for oocyte (or embryo) cryopreservation. And also, some patients who lived in province far from our institution chose ovary tissue preservation. Of course, before made their mind, all patients were given information about the rate of conception after treatment of breast cancer. And also, we told them the risk that ovarian reserve could be decreased by ovariectomy and the risk of dysfunction of ovary tissue after thawing, especially on diminished ovarian reserve patients. In addition, we have explained about contamination of minimum residual disease. Especially for BRCA 1/2 mutation carrier patients (mainly triple negative type breast cancer patients), the fact that the ovary tissue had potential risk of ovarian cancer was informed.

### Data collection

Drawing from clinical records, data were collected on the following epidemiological parameters for breast cancer patients: the patient’s age, body mass index (BMI), marital status, phase of menstruation (follicular or luteal) at the time of operation, level of anti-Müllerian hormone (AMH) at the time of first visit to our hospital, phenotype of breast cancer, whether the patient received treatment for breast cancer before ovary tissue cryopreservation, and laparoscopic surgical procedure (multiple-port laparoscopy or single-incision laparoscopy). Serum AMH levels were measured using a commercial assay kit (AMH Gen II ELISA, BECKMAN COULTER, Brea, CA, USA) according to the manufacturer’s protocol. The detection limit of this kit was 0.16 ng/ml.

### Surgical techniques

Of the 27 patients examined, 16 underwent multiple-port laparoscopy and 11 underwent single-incision laparoscopy. During laparoscopy, a 5-mm scope (HOPKINS 30 K 260468A, Image1 H3-Z K2220055-3; KARL STORZ, Tuttlingen, Germany) was used to secure the field of vision. An incision was made in the umbilical region using a closed approach for entry into the peritoneal cavity. A 5-mm trocar (ENDOPATH EXCEL Trocars; ETHICON Endo-Surgery, JOHNSON AND JOHNSON, NJ, USA) was inserted into the umbilical region to accommodate the 5-mm scope. Then, 5-mm and 11 mm trocars were inserted into the left inguinal and midline regions of the abdominal wall under the laparoscope. The E-Z ACCESS (FF07; HAKKO, Nagano, JAPAN) and LAP PROTECTOR (FF0707; HAKKO, Nagano, JAPAN) were used for single-incision laparoscopy. A 2 cm incision was made at edge of the umbilicus and the open method was used to place the E-Z ACCESS so it would reach the peritoneal cavity. Three 5-mm ports (E-Z trocar smart insertion S701 V12, E-Z trocar smart insertion S701 CV12; HAKKO, Nagano, JAPAN) were inserted into the E-Z ACCESS. Hemilateral ovariectomy for ovarian cryopreservation was performed during multiple-port or single-port laparoscopic surgery. After minimal thermo-coagulation using Bipolar (Bicoag E23-0003 3600, AMCO, Tokyo, Japan) to prevent hemorrhaging, the hemilateral whole ovary was extracted by severing the ovarian arteries and veins and the associated ligaments of the ovary using scissor forceps. After the hemilateral whole ovary was extracted, sites of bleeding were cauterized tightly to stop bleeding, including ovarian vessels and ligaments. Then extracted whole ovary was placed in saline solution at 37 °C and immediately transported to a tissue culture room.

### Ovarian tissue preparation

Isolated whole ovaries were cut into two identical halves using a surgical knife. Then the medulla was isolated from the rest of the ovary using surgical scissors. Following isolation of the medulla, the ovarian cortical tissue was divided into segments (approx. 10 mm × 10 mm × 1 mm) using a surgical knife. The pieces were used for ovarian tissue cryopreservation. This process was performed in a petri dish (Bateriological Petri Dish 60x15 mm, FALCON 351007; Becton, Dickinson and Company, NJ, USA) containing mHTF culture medium (with Hepes; KITAZATO Biopharma Co., Ltd. Shizuoka, Japan) supplemented with 10 % SSS (Serum Substitute Supplement not for Injection, IRVINE, Santa Ana, CA, USA) pre-warmed to 37 °C. Following vitrification [7, 8], ovarian tissue sections were stored in liquid nitrogen for fertility preservation.

### Oocyte extraction

The process of oocyte extraction was carried out in two-steps. First, oocytes were extracted by follicle fluid aspiration from ovarian tissue using a 22-gauge needle (JS-NS2232SP; JMS Co. Hiroshima, Japan) and a 2.5 ml syringe (SS-02Sz; TERMO Co., Tokyo, JAPAN). Second, under a stereo microscope (M165C-SM; LEICA, Solms, Germany) oocytes were identified and collected from the medium using a 200 μl pipet (Calibrated pipet 2-000-200; DRUMMOND SIENTIFIC COMPANY, PA, USA) for ovarian tissue preparation. We collected the oocyte as many as possible by aspiration from all follicles including small antral follicles without measurement of follicle diameter.

### In vitro maturation of oocytes

IVM of oocytes extracted from ovarian tissue germinal vesicles was carried out using IVM medium (IVM system 82214010A; ORIGIO, Malov, Denmark) containing 10 % SSS (Serum Substitute Supplement not for Injection, IRVINE, Santa Ana, CA, USA) and 37.5 mIU/ml of FSH (Gonalef; MERCK SERONO, Darmstadt, Germany). After an incubation of 24–48 h at 37 °C, atmosphere of 5 % CO_2_, and 5 % O_2_ in a high humidity culture, we assessed oocyte maturation. Oocyte maturation was determined microscopically by observing the extrusion of the first polar body into the perivitelline space, which denotes maturation to the metaphase II (MII) stage. Conversely, oocytes that exhibited a morphological change or turned brownish were determined to be degenerated oocytes.

### Statistical analysis

Data analysis was performed using the Statistics Package for Social Sciences (SPSS 11.0, Chicago, IL). To investigate the correlations between patient AMH levels, age, BMI, ovarian tissue volume, and the number of extracted oocytes, we used a non-parametric Spearman’s correlation coefficient by rank test. To investigate the impact of various epidemiological factors (especially phase of menstrual cycle) on the number of oocytes, survival rate (total oocytes extracted minus the number of degenerated oocytes / total oocytes extracted), and maturation rate (MIIoocytes / total extracted oocytes minus the degenerated oocytes), we used a non-parametric Mann-Whitney’s *U* test; *P* <0.05 was considered statistically significant.

## Results

The number of young breast cancer patients who underwent procedures for ovarian tissue cryopreservation was 27. The mean age of patients was 33.7 (±3.8) years. There were no polycystic ovary syndrome patients, and all patients had regular menstrual cycles prior to treatment. Ten patients were married, and 17 were single. Of note, 20 had never been pregnant while 7 had experienced pregnancy. The average body mass index (BMI) was 21.4 (±3.6). Fifteen patients underwent surgery during the follicular phase of the menstrual cycle (before ovulation), 10 patients underwent surgery during the luteal phase of the menstrual cycle (post-ovulation), and for the remaining 2 patients the menstrual stage was unknown. None of the patients were in the ovulation phase. The patients’ molecular subtype of breast cancer were as follows: 5 were luminal A, 20 were luminal B, and one was basal-like type; therefore, one was HER 2 type. The average AMH level was 3.5 (±2.1) ng/ml. The average number of extracted oocytes was 8.3 (±6.1), and the number of ovarian tissue pieces obtained was 16.3 (±5.1). Two of the patients underwent chemotherapy and one underwent surgery prior to ovarian tissue cryopreservation. Sixteen underwent multiple-port laparoscopy and eleven underwent single-port laparoscopy for ovarian tissue cryopreservation. Detailed data regarding individual patients is shown in Table 1.

**Table 1:** Characteristics of breast cancer patients who desired to undergo ovarian tissue cryopreservation

No.	Age (years old)	Marital Status	Past pregnancies	BMI	Phase of mentruation (Day)	AMH (ng/ml)	No. of oocytes	No. of ovarian tissues	Procedure of laparoscopy	Molecular subtype	Pretreatment
1	34	Single	None	23.7	F (6)	4.2	17	18	Multiple	A	
2	33	Married	None	25	L (22)	6.90	11	12	Multiple	B	FEC*4 DOC/HCN*4
3	32	Single	None	19	F (6)	3.16	12	28	Multiple	B	
4	39	Single	None	20.8	F (3)	3.98	5	16	Multiple	A	Radiation
5	39	Married	None	19.2	F (4)	1.57	4	18	Multiple	B	
6	29	Married	None	23.1	F (3)	5.82	19	26	Multiple	B	
7	33	Married	1G1P	30.6	F (7)	3.06	2	16	Multiple	B	
8	30	Single	None	23.8	L (22)	6.62	7	15	Multiple	B	
9	36	Single	None	201	F (6)	1.37	7	17	Multiple	B	GnRHa 2y, TAM 5y
10	41	Married	1G0P	32.8	L (17)	0.72	4	9	Multiple	B	
11	36	Single	1G0P	18.2	unknown	1.45	5	20	Multiple	B	
12	35	Single	None	19.4	F (7)	0.87	0	22	Multiple	B	
13	33	Single	None	19.7	L (28)	6.13	26	23	Multiple	B	
14	33	Single	None	19.9	L (25)	2.05	9	18	Multiple	A	
15	34	Married	None	18.9	F (9)	1.09	0	20	Multiple	B	CE*4 DOC/HCN*4
16	32	Single	None	19.5	F (8)	4.35	14	17	Multiple	HER2	
17	34	Single	None	20.1	L (16)	2.21	6	12	Single	Tri neg	
18	37	Married	None	17.7	L (25)	0.7	2	15	Single	A	TAM 2 M
19	36	Married	1G0P	23.9	L (32)	3.03	10	6	Single	B	
20	29	Single	None	19.6	unknown	5.2	6	19	Single	B	
21	33	Married	3G0P	20.2	F (14)	5.31	9	17	Single	B	surgery
22	38	Married	1G0P	24.5	F (8)	0.52	10	8	Single	B	
23	34	Single	None	21.1	F (13)	3.56	2	11	Single	A	
24	36	Single	None	22.3	L (23)	3.82	5	14	Single	B	
25	34	Single	None	18.6	F (3)	5.51	9	14	Single	B	
26	25	Single	None	17.6	F (6)	7.73	17	14	Single	B	
27	25	Single	1G0P	19	L (29)	2.54	8	16	Single	B	

*F* Follicular phase, *L* Luteal phase, *Multiple* multiple port surgery, *Single* Single port laparoscopy, *A* Luminal A type, *B* Luminal B type, *Tri neg* triple negative type, *TAM* tamoxifen, *FEC* 5-FU and epirubicin and cyclophosphamide, *DOC* docetaxel, *HCN* trastuzumab, *CE* cyclophosphamide and epirubicin, *GnRHa* gonadotropin releasing hormone agonist

**Fig. 1:**
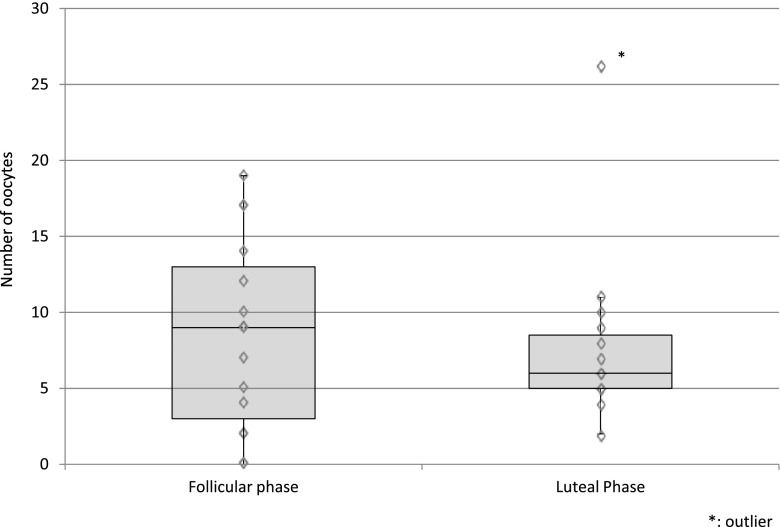
The correlation between the menstrual cycle phase and the number of extracted oocytes. With regard to phase in the menstrual cycle, there was no significant difference between groups in the number of extracted oocytes (*P* = 0.99 non-parametric Mann-Whitney’s *U* test)

**Fig. 2:**
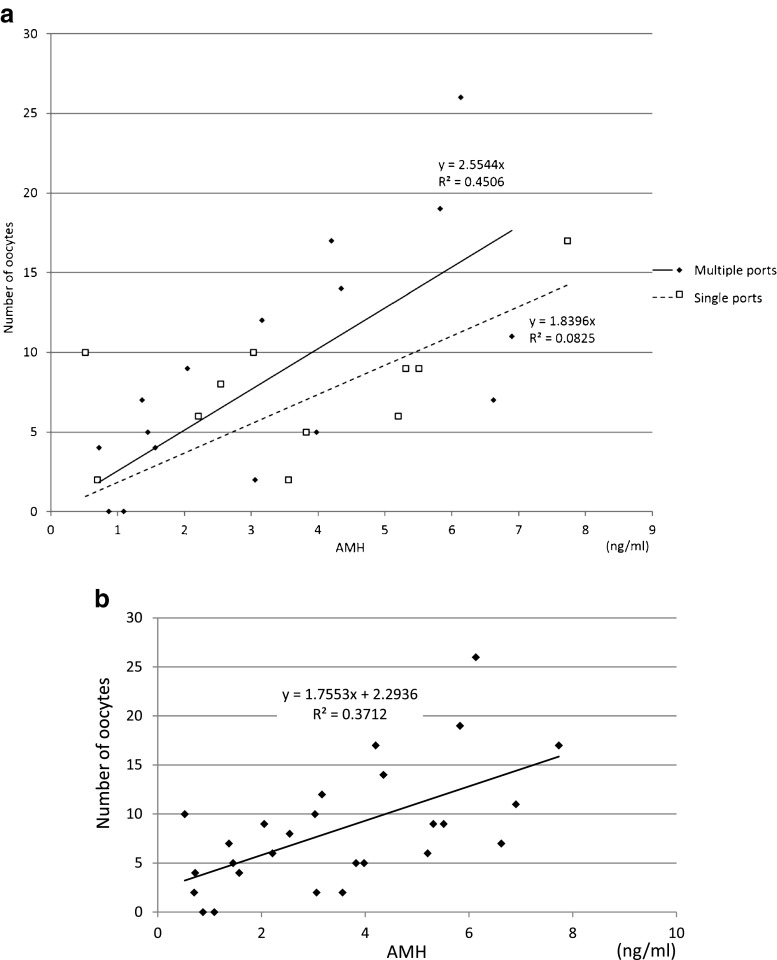
The correlation between patient AMH level and the number of extracted oocytes. **a** With regard to laparoscopic procedure, there was no significant difference between groups in the number of extracted oocytes and AMH levels (*P* = 0.94, 0.88 respectively. non-parametric Mann-Whitney’s *U* test). **b** A strong correlation was observed between AMH levels and the number of extracted oocytes (correlation coefficient: 0.60, Spearman’s correlation coefficient by rank test)

**Fig 3:**
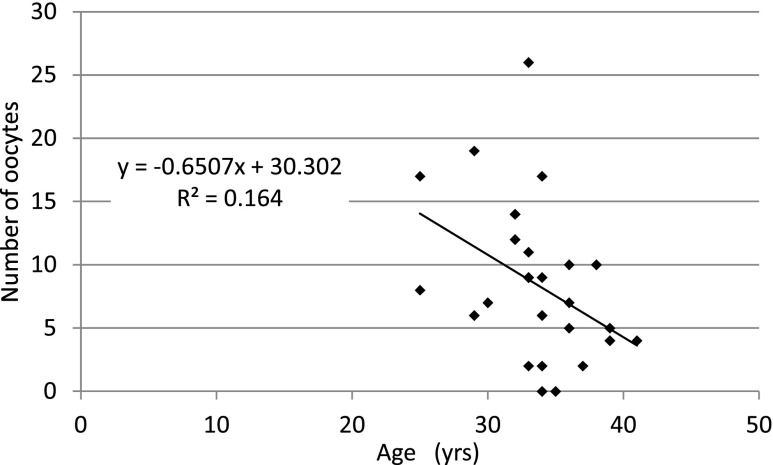
The correlation between age and the number of extracted oocytes. A correlation was observed between age and the number of extracted oocytes (correlation coefficient: −0.48, Spearman’s correlation coefficient by rank test)

With regard to the phase in the menstrual cycle (*P* = 0.99) (Fig. 1), laparoscopic procedure (*P* = 0.94) (Fig. 2a), marital status (*P* = 0.47), previous pregnancy experience (*P* = 0.68), molecular subtype of breast cancer (comparing only Luminal A to B) (*P* = 0.52), there was no significant difference between the groups in the number of oocytes extracted. In Fig. 2a we show that there was no significant difference in AMH levels with regard to laparoscopic procedure (*P* = 0.88) (Fig. 2a). And also, there was no significant difference in terms of the survival and maturation rates between the follicular phase and the luteal phase (Table 2). Patients 11 and 20 were excluded from Table 2 because their phase in the menstrual cycle was unknown at the time of surgery. Furthermore, we found that the patient’s AMH level and the number of extracted oocytes was well-correlated (coefficient of correlation: 0.60) (Fig. 2b). Also, the age at which the ovarian tissue was cryopreserved and the number of extracted oocytes was correlated (coefficient of correlation: −0.48) (Fig. 3). However, we did not find any correlation between BMI or the number of ovarian tissue segments and the number of extracted oocytes (coefficient of correlation: 0.08, 0.10, respectively).

**Table 2:** The comparison of the IVM results from the follicular and luteal phases

Phase	No.	Total	MII	MI	GV	Deg.	SR^a^ (%)	MR^b^ (%)
Follicular	1	17	7	4	2	4	76.5	53.8
	3	12	1	4	4	3	75	11.1
	4	5	1	4	0	0	100	20
	5	4	3	0	0	1	75	100
	6	19	4	6	3	6	68.4	30.8
	7	2	0	2	0	0	100	0
	9	7	3	2	0	2	71.4	60
	12	0	-	-	-	-	-	-
	15	0	-	-	-	-	-	-
	16	14	6	3	2	3	78.6	54.5
	21	9	6	0	2	1	88.9	75
	22	10	3	3	1	3	70	42.9
	23	2	1	0	0	1	50	100
	25	9	7	1	1	0	100	77.8
	26	17	9	3	2	3	82.4	64.3
	mean	8.5(±6.3)	3.9(±2.8)	2.5(±1.9)	1.3(±1.3)	2.1(±1.8)	79.7(±14.6)	53.1(±31.6)
	No.	Total	MII	MI	GV	Deg.	SR (%)	MII rate (%)
Luteal	2	11	4	5	1	1	90.9	40.0
	8	7	4	2	1	0	100	57.1
	10	4	2	1	0	1	75	66.7
	13	26	13	7	1	5	80.8	61.9
	14	9	2	3	1	3	66.7	33.3
	17	6	3	2	0	1	83.3	60
	18	2	0	0	0	2	0	-
	19	10	2	5	1	2	80	25
	24	5	2	1	0	2	60	66.7
	27	8	0	5	0	3	62.5	0
	Mean	8.8(±6.6)	3.2(±3.7)	3.1(±2.3)	0.5(±0.5)	2(±1.4)	69.9(±27.5)	45.6(±22.9)
	*P* value	0.99	0.37	0.53	0.16	0.98	0.51	0.66

Values are expressed mean ± SD

*No*. patient number, *Deg*. degeneration oocyte, *SR* survival rate, *MR* maturation rate

^a^survival rate = total oocytes extracted minus the number of degenerated oocytes / total oocytes extracted)

^b^maturation rate = MIIoocytes / total extracted oocytes minus the degenerated oocytes)

Qualitative data were expressed as numbers and compared using the non-parametric Mann-Whitney’s *U* test. (*P* < 0.05)

## Discussion

In a typical IVM cycle, the number of available oocytes is the most significant factor associated with a successful procedural outcome in normo-ovulatory patients [12]. Therefore, it is necessary to elucidate the factors associated with the number of oocytes obtained from the “combined procedure” for oncofertility treatment. In a previous report, researchers found that patients’ AMH levels and the number of extracted oocytes were well-correlated in the 80 patient normo-ovulately group (mean age; 31.5 ± 5, mean AMH level; 2.7 ± 2.5 ng/ml, mean BMI 22 ± 2) and the 20 patient polycystic ovary syndrome group (mean age; 29 ± 4, mean AMH level; 8.4 ± 4 ng/ml, mean BMI 23 ± 2) [13]. In both groups, there was a strong, positive correlation between patients’ AMH levels and the number of extracted oocytes. There was also a negative correlation between age and the number of extracted oocytes [13]. These previous findings are congruent with the current study. Therefore, we conclude that the number of oocytes extracted from cryopreserved ovarian tissue is well-correlated with the breast cancer patients’ AMH levels and age at the time of surgery.

Previously it was shown that the stage of menstruation (follicular or luteal phase) did not significantly affect either the number of oocytes collected, the total number of MII oocytes, or the maturation rate of oocytes in a hormone-stimulated, follicle stimulating hormone (FSH) and human chorionic gonadotropin (hCG), IVM cycle [14]. Furthermore, consistent with the present study, the researchers reported that the number of immature oocytes that could be retrieved from a partially excised ovary did not depend upon the menstrual phase when using the “combined procedure” [11]. In a study of an unstimulated IVM cycle, oocytes were retrieved in both the luteal and the follicular phases [15]. Likewise, collection during the follicular or luteal phase using the “combined procedure” produced similar results and was not significantly different. In a nonhuman primate (baboon) IVM study, there was a maturation rate >40 % using oocytes extracted from the ovary during the luteal phase [16]. Whereas, a bovine IVM study revealed that the number of oocytes retrieved from the luteal phase ovary was less than number of oocytes retrieved from the early follicular phase; although, it should be noted that there was no significant difference in terms of the maturation rate or the number of degenerated oocytes [17]. Though the data support the efficacy of the “combined procedure” during any menstrual phase, a larger-scale human study is needed to clarify the true efficacy of “combined procedure” during the luteal phase of menstruation. This study should highlight the fertility and pregnancy rates of women after treatment.

In the present study, 2 patients had undergone chemotherapy, and another 2 patients were undergoing gonadal suppression therapy using gonadotropin releasing hormone agonist (GnRHa) with tamoxifen. As mentioned above, the chemotherapy containing cyclophosphamide for breast cancer has an adverse effect on ovarian reserve. Although we could not determine the effect of chemotherapy with only four patients, we can extract oocytes in accordance with the AMH levels in similar patients who undergo treatment for breast cancer. A larger scale study is needed to assess the effect of chemotherapy and GnRHa on the outcome of the “combined procedure”.

The number of extracted oocytes in this study was 8.2 (±6.0) (range; 0–26). According to previous reports, the range of the collected oocytes from a bilateral ovaries isolation and the “combined procedure” was “0 to 10” and “1 to 4”, respectively [18, 19]. Consequently, we conclude that our procedure for oocyte collection was appropriate. In comparison to previous reports [18, 19], our method is safer and is a minimally invasive technique; our method does not require trans-vaginal or trans-abdominal oocyte pick up. Although single-port laparoscopy is a more difficult surgical procedure than multiple-port laparoscopy, postoperative recovery is faster for single-port laparoscopy because it requires only one small incision [18]. In present study, there was no significant difference between the surgical procedure type and the number of oocytes that could be extracted. Therefore, the type of surgical procedure chosen depends on the condition of adhesion, that is, with or without endometriosis [18].

In conclusion, the outcome of the “combined procedure” depends upon the patient’s AMH level and age. Although random-start controlled ovarian hyperstimulation was reported recently as successful emergency fertility preservation method [20–22], the method is not yet widespread. Until it becomes more widely available, the “combined procedure” can be used in any phase of the menstrual cycle to preserve the fertility of breast cancer patients. In addition, it is need to clarify the effect of “combined procedure” including assessment of fertility rate and live birth rate based on more large scale study.
